# Potential reduction in female sex workers’ risk of contracting HIV during coronavirus disease 2019

**DOI:** 10.1097/QAD.0000000000002943

**Published:** 2021-05-10

**Authors:** Fortunate Machingura, Sungai T. Chabata, Joanna Busza, Gracious Jamali, Memory Makamba, Jeffrey Dirawo, Raymond Yekeye, Owen Mugurungi, Primrose Matambanadzo, Frances Mary Cowan

**Affiliations:** a Centre for Sexual Health and HIV/AIDS Research Zimbabwe, Harare, Zimbabwe; b Centre for Evaluation, London School of Hygiene and Tropical Medicine, London, UK; c National AIDS Council of Zimbabwe; d Ministry of Health & Child Welfare, Harare, Zimbabwe; e Department of International Public Health, Liverpool School of Tropical Medicine, Liverpool, UK.

## Abstract

Female sex workers’ livelihoods in Zimbabwe have been severely impacted by the coronavirus disease 2019 pandemic due to closure of entertainment venues. Competition over fewer clients has reduced ability to negotiate condom use. At the same time as partner numbers have decreased, frequency of reported condomless sex has not increased, suggesting potential reduction in overall HIV and sexually transmitted infection risk and an opportunity for programmes to reach sex workers with holistic social and economic support and prevention services.

The global coronavirus disease 2019 (COVID-19) pandemic has disrupted economies across the world, disproportionately threatening the livelihoods of people working in the informal sector with low-wage jobs [1]. These include sex workers, who are further marginalized due to the criminalization of sex work [2]. Reports from diverse regions suggest sex workers continue to work despite restrictions to survive, but struggle to find clients and experience increased vulnerability to stigma, violence and police harassment [3].

In Zimbabwe, Sisters with a Voice is a nationally scaled HIV prevention and treatment programme for sex workers that reaches over 26 000 female sex workers (FSW) annually with social and clinical services [4]. During Zimbabwe's national lockdown (April–October 2020), we collected data from FSW visiting our two largest clinics in Harare and Bulawayo on their client numbers, earned income, work conditions and condomless sex, which we compared with our most recent representative data from Respondent Driven Surveys (RDS) conducted in these sites in 2017.

We found 90% FSW attending these clinics reported reduced client numbers. In 2017 RDS, *weekly* client numbers averaged 14 in Harare and eight in Bulawayo but since lockdown, FSW reported mean *monthly* client numbers of nine and three, respectively. Of these, FSW reported condomless sex with two of nine clients (Harare) and one of three (Bulawayo) following lockdown compared with 2 of 52 and 1 of 32 in 2017, but absolute numbers of condomless partners did not increase. Anecdotally, sex workers report that closure of entertainment venues, restrictions on mobility, and male clients’ fear of contracting COVID-19 have significantly reduced earnings. When FSW do procure a client, they are less likely to negotiate condom use or high fees, and are more willing to accept condomless sex and exchange sex for food.

Restrictions in Zimbabwe have constrained FSW ability to work, negotiate condom use or refuse clients, increasing their social and economic marginalization. However, it is possible that a reduction in overall client numbers without an accompanying increase in condomless sex has not increased their risk of HIV and STI, and possibly decreased it. The *Sisters* programme has addressed FSW precarious survival at this time by offering psychosocial support and livelihood assistance; for example, by facilitating self-help groups to set up shared savings and income support schemes, including making facemasks to sell. It is imperative to address FSWs’ needs holistically as well as reinforce HIV prevention messages to take advantage of a possible reduction in HIV risk by ensuring its sustainability.

## Acknowledgements

### Conflicts of interest

There are no conflicts of interest.
